# REW-DETR: A Lightweight Real-Time Detection Transformer for Bridge Surface Defect Detection

**DOI:** 10.3390/s26206401

**Published:** 2026-10-10

**Authors:** Xiaohui Cheng, Yonghong Pang, Yanjun Wang, Yun Deng, Qiu Lu

**Affiliations:** 1 College of Computer Science and Engineering, Guilin University of Technology, Guilin 541004, China; cxiaohui@glut.edu.cn (X.C.); 2120241292@glut.edu.cn (Y.P.); 2002078@glut.edu.cn (Y.D.); 2002021@glut.edu.cn (Q.L.); 2 College of Information Engineering, Guilin College, Guilin 541006, China; 3 Guangxi Key Laboratory of Embedded Technology and Intelligent System, Guilin 541004, China

**Keywords:** bridge surface defect detection, UAV inspection, real-time object detection, lightweight transformer detector, channel attention, bounding box regression

## Abstract

Timely detection of bridge surface defects is important for structural safety assessment and inspection efficiency. To address defect scale variation, complex background interference, and the high computational cost of RT-DETR-R50, this study proposes REW-DETR, a lightweight bridge surface defect detector. REW-DETR adopts a coordinated lightweight design integrating efficient feature extraction, multi-level channel recalibration, and localization optimization. RepNCSPELAN4–ADown is employed to reconstruct the backbone and fusion stages, ECA is embedded at the P3–P5 feature levels to enhance channel-wise feature discrimination, and Wise-IoU v3 optimizes bounding-box regression without inference-time overhead. On the test set of the bridge surface defect dataset, REW-DETR achieved Precision, Recall, mAP_50_, and mAP_50:95_ values of 87.0%, 70.4%, 73.7%, and 42.4%, respectively. These values exceeded those of RT-DETR-R50 by 3.8, 5.5, 4.0, and 4.5 percentage points, respectively. Meanwhile, the parameters and FLOPs were reduced by approximately 53.3% and 60.6%, respectively, while FPS increased from 51.2 to 70.8. After dataset-specific retraining on the bridge-defects-detection dataset from Roboflow Universe, REW-DETR achieved a Recall of 58.0% and an mAP_50_ of 55.4% while retaining a lightweight configuration. These results demonstrate an improved accuracy–efficiency trade-off and motivate further evaluation of REW-DETR for UAV-assisted bridge inspection, while onboard inference performance remains to be validated.

## 1. Introduction

Bridges serve as fundamental components of transportation infrastructure, and their structural condition is closely associated with operational safety and maintenance planning. During long-term service, repeated traffic loading, environmental exposure, and material degradation may cause various surface defects, including cracks, corrosion, concrete deterioration, voids, and water leakage. Although these defects are initially localized at the surface, their progressive development may accelerate structural deterioration, increase maintenance requirements, and potentially threaten the safety of bridge operation. Therefore, accurate and efficient identification of bridge surface defects is essential for condition assessment, preventive maintenance, and intelligent bridge health management [1,2].

Conventional bridge inspection practices largely depend on manual visual examination and expert experience, making the process time-consuming and susceptible to subjective judgments, especially when inspecting large-scale structures or inaccessible areas. The emergence of unmanned aerial vehicles (UAVs), high-resolution imaging sensors, and image processing techniques has provided new possibilities for non-contact bridge inspection. Compared with traditional approaches, UAV-based image acquisition enables flexible data collection from complex structural components, including bridge undersides, high piers, and regions above water, thereby improving inspection efficiency and reducing operational risks [2].

In recent years, deep learning-based object detection approaches have been increasingly explored for automated bridge surface defect identification. Among these methods, YOLO-series detectors have received extensive attention due to their favorable trade-off between detection accuracy and inference speed. Existing studies have improved YOLO-based bridge defect detectors by incorporating feature enhancement modules, attention mechanisms, and regression optimization strategies to achieve more reliable defect recognition under complex conditions [3]. However, most YOLO-based methods still rely on manually designed post-processing procedures and convolution-based feature extraction pipelines, which may limit their adaptability to complex bridge inspection scenarios requiring both accurate localization and efficient deployment. Meanwhile, RT-DETR [4], inspired by the end-to-end detection paradigm of DETR [5], introduces transformer-based set prediction into real-time object detection. Through the hybrid encoder and IoU-aware query selection mechanism, RT-DETR removes the dependence on non-maximum suppression (NMS) during inference and achieves competitive detection performance with real-time efficiency.

However, the direct application of RT-DETR-R50 to bridge surface defect inspection remains challenging. First, complex surface conditions, including concrete textures, illumination variations, shadows, and water stains, may generate responses similar to actual defects and reduce detection reliability. Second, bridge defects exhibit considerable scale diversity, ranging from slender cracks and small voids to large-area concrete deterioration, making multi-scale feature representation difficult. Third, although RT-DETR-R50 provides strong detection capability, its relatively large backbone and computational requirements hinder deployment on UAV platforms and other resource-limited devices. Therefore, improving defect representation capability while reducing model complexity is essential for bridge surface defect detection in practical inspection scenarios. Accordingly, this study develops REW-DETR, a lightweight RT-DETR-R50 variant for multi-class bridge surface defect detection. Its design coordinates modifications to lightweight feature extraction and cross-scale fusion, multi-level channel recalibration, and bounding-box regression optimization to jointly address model complexity, multi-scale defect representation, and localization quality.

The principal contributions of this work are as follows:

(1) The backbone and cross-scale feature-fusion stages of RT-DETR-R50 are redesigned using RepNCSPELAN4–ADown and RepNCSPELAN4-based fusion blocks to reduce computational complexity while retaining hierarchical multi-scale feature representations.
(2) ECA is deployed across the P3, P4, and P5 feature outputs to provide lightweight channel recalibration at different semantic levels and strengthen defect-related feature discrimination under complex bridge backgrounds.
(3) Wise-IoU v3 is integrated into the training-stage localization objective to optimize bounding-box regression for defects with irregular or ambiguous boundaries without modifying the inference structure or increasing inference-time complexity.

The remainder of this paper is organized as follows. Section 2 reviews vision-based bridge defect detection, one-stage detectors, real-time Transformer-based detectors, and relevant model-improvement techniques. Section 3 presents the architecture and principal components of REW-DETR. Section 4 describes the datasets, implementation settings, evaluation metrics, and experimental results. Section 5 summarizes the main findings, discusses the limitations of the present study, and outlines directions for future work.

## 2. Related Work

### 2.1. Vision-Based Bridge Defect Detection

Visual sensing offers a non-contact means of recording surface conditions and has become an important source of information for bridge inspection and condition assessment. Image data can preserve the location, appearance, and spatial extent of visible deterioration, supporting the automated recognition of cracks, spalling, corrosion, water leakage, and other surface defects. The review by Luo et al. [6] summarized recent progress in computer vision-based bridge inspection and monitoring, particularly in surface damage identification and automated condition assessment.

Earlier vision-based inspection systems generally relied on handcrafted descriptors, image thresholding, edge extraction, and conventional machine-learning classifiers. Their performance was often sensitive to illumination, surface texture, viewing distance, and camera angle because the selected features had limited adaptability to changes in imaging conditions. Deep convolutional networks reduced this dependence on manually designed representations by learning task-related features directly from images. Qiao et al. [7], for example, applied a deep convolutional network with an attention module to bridge damage detection and reported improved recognition under complex visual conditions.

Advances in UAV imaging and mobile vision systems have also expanded the spatial coverage of bridge inspection. Images can now be collected from bridge undersides, elevated piers, and areas above water that are difficult or hazardous to access using conventional inspection equipment. Recent reviews indicate that the combination of computer vision, deep learning, and mobile sensing has improved the automation and consistency of bridge inspection procedures [8,9]. Nevertheless, images acquired in practical environments still contain substantial illumination variation, irregular backgrounds, weak defect boundaries, and large differences in defect size. These conditions make it difficult to achieve both reliable multi-class detection and efficient inference, particularly when the detector is intended for UAV-assisted or resource-constrained applications.

### 2.2. One-Stage Object Detectors for Bridge Defect Detection

One-stage detectors predict object categories and bounding boxes directly from dense feature maps, avoiding the separate proposal-generation stage used in two-stage frameworks. Their relatively simple inference pipelines and favorable runtime efficiency have made them widely used in bridge surface defect detection. Within this group, YOLO-based models have received particular attention, and most recent improvements have focused on feature enhancement, multi-scale fusion, lightweight network design, and bounding box regression.

For multi-class bridge defect detection, several studies have modified YOLO architectures to better accommodate differences in defect appearance and scale. Chen et al. [10] proposed RAS-YOLO11 by integrating RepN_CAA, ADown, and A-SlimNeck into YOLO11n. Pruning and quantization were further incorporated to support edge deployment. This work reflects a broader shift in bridge inspection research from accuracy-oriented model modification toward a joint consideration of detection performance, computational complexity, and deployment efficiency.

Other studies have concentrated on feature representation in visually complex scenes. Li et al. [11] developed DCNA-YOLO, in which a multi-branch coordinate attention mechanism and deformable convolution were combined to improve the representation of irregular surface defects. BD-YOLOv8s introduced ODConv, CBAM, and CARAFE to strengthen dynamic feature extraction and feature reconstruction [12]. Enhanced YOLOv3 incorporated SENet, spatial pyramid pooling, and CIoU loss to improve apparent bridge damage detection under complex backgrounds [13]. Similarly, an improved YOLOv8n detector jointly optimized attention, feature fusion, upsampling, and localization loss for bridge crack detection [14].

YOLO-based frameworks have also been extended beyond bounding box detection. The YOLOv8-AFPN-MPD-IoU model was developed from YOLOv8s-Seg and combined AFPN with MPD-IoU to support bridge crack recognition, instance segmentation, and geometric measurement [15]. These studies demonstrate the flexibility of one-stage detectors for different bridge inspection tasks. However, many existing methods still depend on NMS-based post-processing, and their performance may remain sensitive to complex surface textures, large variations in defect scale, and demanding localization conditions. These limitations motivate the investigation of end-to-end detectors that can retain real-time efficiency while providing stronger multi-scale representation and localization capability.

### 2.3. Transformer-Based Real-Time Detectors

The introduction of Transformer architectures into computer vision has broadened the design space of object detectors [16]. In contrast to convolutional networks, which build visual representations mainly through local receptive fields, self-attention allows interactions to be established across spatially separated image regions. DETR [5] subsequently formulated object detection as a set-prediction problem and removed the conventional combination of anchor design and NMS-based post-processing. However, the original DETR required a long training schedule and did not provide the inference efficiency needed for real-time applications.

RT-DETR [4] retained the end-to-end set-prediction framework while improving efficiency through a hybrid encoder and IoU-aware query selection. Later studies examined several aspects of this architecture. RT-DETRv2 introduced a collection of training and implementation refinements to establish a stronger real-time detection baseline [17]. DEIM addressed convergence and matching quality through Dense O2O matching and Matchability-Aware Loss, thereby improving the use of candidate predictions during training [18]. These developments indicate that recent DETR-based research has moved beyond basic real-time inference toward better convergence, matching stability, and accuracy–efficiency trade-offs.

RT-DETR has also been adapted to defect inspection in bridges, pavements, and concrete structures. UCRT-DETR combined a lightweight backbone with PKI Block and CAA for robotic inspection of underwater bridge-pier cracks [19]. EMAFG-RTDETR modified RT-DETR-R18 with a lightweight multi-scale attention mechanism and localization-loss optimization for UAV-based concrete defect detection [20]. Pavement-DETR enhanced small-defect representation through attention, multi-scale feature fusion, and combined localization losses in UAV pavement imagery [21]. RAI-DETR introduced receptive-field attention, feature assignment, and Inner-IoU-based localization optimization for real-time concrete crack detection [22].

Efficiency-oriented modifications have received increasing attention because infrastructure inspection models may eventually operate on UAVs or edge devices. EF-RT-DETR redesigned the backbone and feature-fusion components to reduce model complexity while improving pavement-distress detection [23]. DSC-RTDETR combined a YOLOv11 backbone with the RT-DETR decoder and introduced DSConv and BiFormer attention for concrete crack detection [24]. Other recent methods have similarly explored lightweight backbones, attention modules, multi-scale fusion, and regression-loss redesign for surface-defect and road-crack detection [25,26].

Overall, existing RT-DETR-based infrastructure inspection methods emphasize different aspects of defect detection. UCRT-DETR combines a lightweight backbone with PKI Block and CAA for underwater bridge-pier crack inspection, whereas EF-RT-DETR redesigns the backbone and feature-fusion components to reduce model complexity. EMAFG-RTDETR and Pavement-DETR combine feature enhancement with localization-related optimization. RAI-DETR emphasizes receptive-field attention, feature assignment, and Inner-IoU-based regression, while DSC-RTDETR combines a YOLOv11 backbone with DSConv and BiFormer attention. In comparison, REW-DETR targets multi-class bridge surface defects by coordinating lightweight backbone and feature-fusion reconstruction, multi-level channel recalibration at P3–P5, and training-stage bounding-box regression optimization within RT-DETR-R50. These differences show that REW-DETR places particular emphasis on jointly balancing detection accuracy and model complexity through coordinated modifications at the feature-extraction, feature-enhancement, and regression-optimization stages. The lightweight design, feature enhancement, and bounding-box regression strategies most relevant to this study are further discussed in the following subsection.

### 2.4. Lightweight Design, Attention Mechanisms and Bounding Box Regression Optimization

The deployment of bridge surface defect detection models on UAV platforms, mobile devices, and edge computing systems requires a careful balance between detection accuracy and computational efficiency. Although deeper and more complex networks can improve feature representation, the associated increases in parameters, FLOPs, and inference latency may restrict their practical application in resource-limited inspection scenarios. Consequently, lightweight architectures, feature enhancement mechanisms, and localization optimization have become important strategies for improving detection models.

Lightweight network design aims to reduce computational redundancy while maintaining sufficient representation capability. RepNCSPELAN4 introduced in YOLOv9 exploits re-parameterized multi-branch feature extraction during training and converts the structure into a more efficient form during inference, achieving a favorable balance between accuracy and efficiency [27]. For bridge defect detection, lightweight backbone design should not only decrease model complexity but also preserve hierarchical feature representation, since defects such as slender cracks, small-scale cavities, corrosion regions, and large-area concrete deterioration exhibit substantial differences in spatial characteristics.

Attention mechanisms provide an alternative approach to improve feature selectivity by enhancing informative responses and suppressing irrelevant background activations. In bridge inspection images, surface textures, illumination variations, shadows, and water stains may generate responses similar to actual defects. ECA-Net introduces efficient channel interaction through global average pooling and one-dimensional convolution without dimensionality reduction, providing a lightweight solution with limited parameter overhead and convenient integration [28]. Therefore, lightweight channel attention mechanisms are particularly suitable for improving defect-related feature discrimination under complex visual conditions.

In addition to feature representation, bounding box regression quality directly affects object localization accuracy. Conventional IoU-based losses mainly optimize geometric overlap between predicted and reference boxes, but their effectiveness may decrease when localization errors are large or the overlap is insufficient. Wise-IoU introduces a dynamic focusing strategy that adjusts regression contributions according to sample quality, allowing the model to prioritize more valuable regression samples during optimization [29]. This property is beneficial for bridge defect detection, where irregular boundaries and ambiguous defect regions can increase localization uncertainty.

Taken together, lightweight architectures, attention mechanisms, and bounding box regression losses have all shown value in infrastructure defect detection. However, their coordinated design for lightweight multi-class bridge surface defect detection remains comparatively underexplored, particularly when cross-scale feature representation, localization quality, model complexity, and deployment efficiency must be considered simultaneously. To address this gap, REW-DETR jointly redesigns lightweight feature extraction and multi-scale fusion, multi-level channel enhancement, and localization optimization within the RT-DETR-R50 framework.

## 3. REW-DETR

### 3.1. Overall Architecture

**Figure 1:**
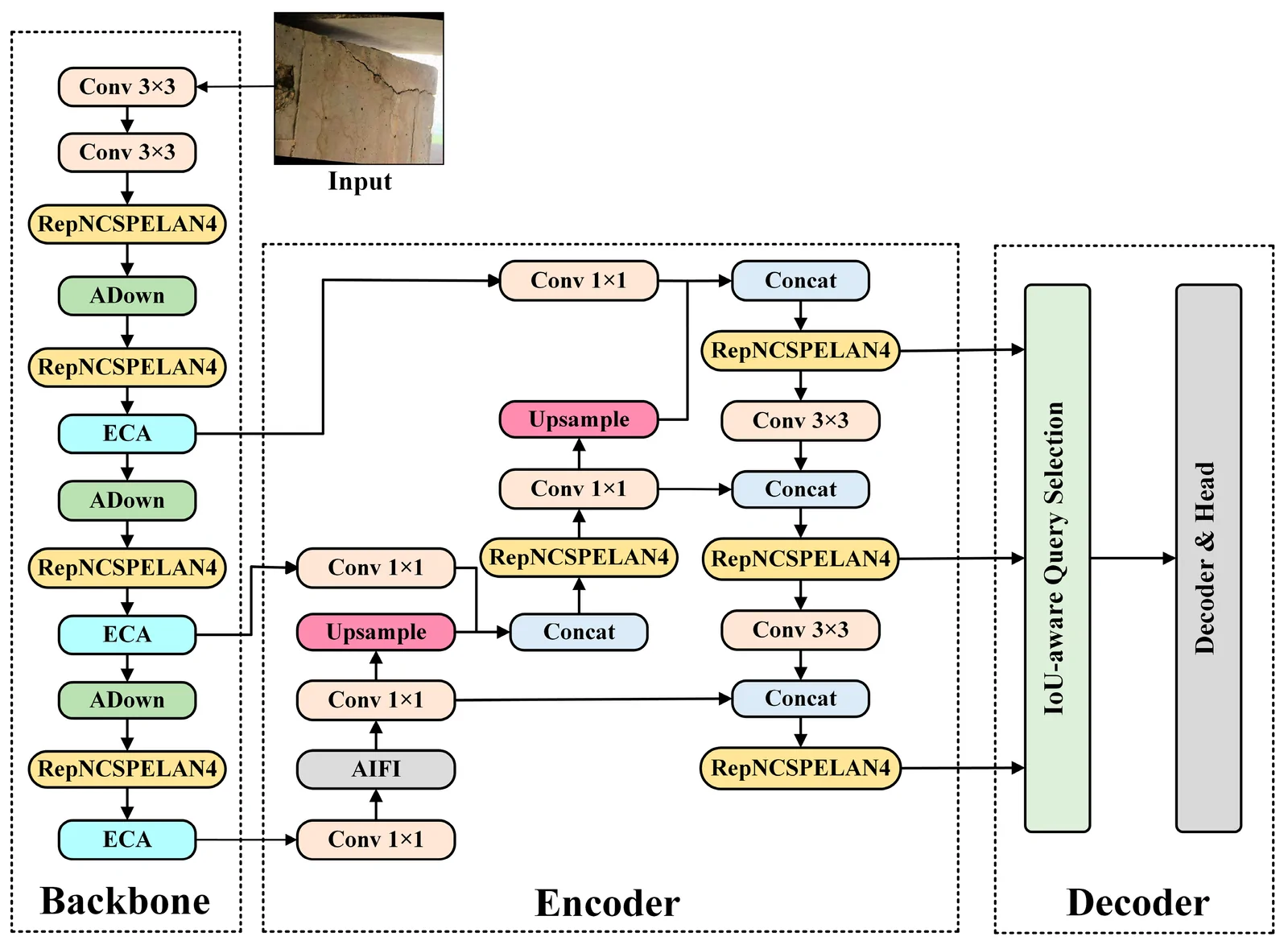
Inference architecture of REW-DETR.

This study proposes REW-DETR, a lightweight end-to-end detector for multi-class bridge surface defect detection, based on RT-DETR-R50. As illustrated in Figure 1, REW-DETR retains the original RT-DETR detection pipeline, including multi-scale feature extraction, hybrid encoding, IoU-aware query selection, and transformer-based decoding. Targeted modifications are introduced into the backbone, hybrid encoder, multi-level feature outputs, and localization objective to improve the accuracy–efficiency balance.

The original ResNet50 backbone is replaced with a lightweight architecture composed of RepNCSPELAN4 and ADown modules [27], which generates the P3, P4, and P5 hierarchical feature maps. ECA modules are inserted after these three backbone outputs to perform multi-level channel enhancement. In the hybrid encoder, the original top-down and bottom-up cross-scale interaction topology is retained, while the RepC3 fusion blocks are replaced with RepNCSPELAN4 modules. The resulting multi-scale representations are then delivered to the query selection module and RT-DETR decoder for defect classification and bounding box prediction.

During training, Wise-IoU v3 replaces the original GIoU term in the localization objective. This modification optimizes bounding box regression without changing the inference graph or introducing additional inference-time complexity. The detailed structures and implementations of the lightweight feature extraction and fusion architecture, multi-level ECA, and Wise-IoU v3 are presented in Section 3.2, Section 3.3 and Section 3.4, respectively.

### 3.2. RepNCSPELAN4-Based Lightweight Feature Extraction and Multi-Scale Fusion

**Figure 2:**
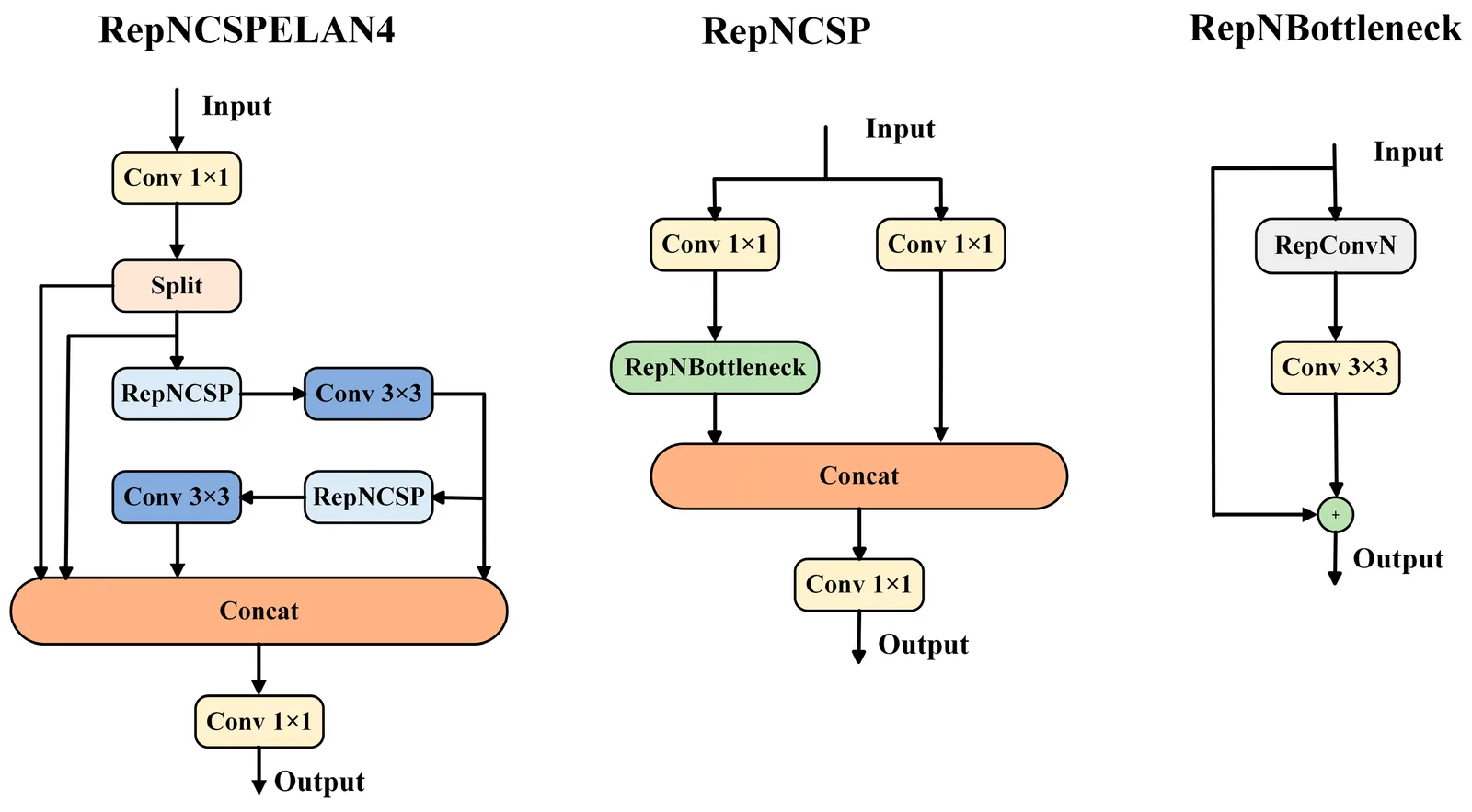
Structural illustration of RepNCSPELAN4 and its internal components.

To improve the feature extraction efficiency of RT-DETR-R50 while maintaining sufficient representation capability, the original ResNet50 backbone is replaced with a lightweight architecture composed of RepNCSPELAN4 and ADown modules adopted from YOLOv9 [27]. Compared with conventional residual convolution structures, the lightweight backbone introduces re-parameterized feature transformation and efficient downsampling to reduce redundant computation while preserving hierarchical feature information. RepNCSPELAN4 blocks are also incorporated into the hybrid encoder to enhance cross-scale feature fusion. The structure of RepNCSPELAN4 and its internal components is illustrated in Figure 2.

RepNCSPELAN4 combines channel partitioning, cross-stage feature propagation, and re-parameterized convolution for efficient feature transformation. As shown in Figure 2, the input feature is first processed by a 1 × 1 convolution and then divided into separate branches along the channel dimension. The main branch performs progressive feature refinement through RepNCSP structures and convolution operations, while the shortcut branch provides a more direct feature propagation path to preserve information during deep feature processing.

After branch-wise transformation, the generated features are concatenated and compressed through a 1 × 1 convolution. This operation integrates information from multiple transformation paths while limiting the redundant computation associated with repeatedly stacking conventional convolution blocks.

Within RepNCSPELAN4, the RepNCSP component adopts a dual-branch structure. One branch performs deeper feature extraction through RepNBottleneck, whereas the other provides a relatively direct information propagation path. RepNBottleneck introduces multiple convolution branches during training to strengthen feature representation. During inference, these branches are equivalently merged into a single convolution operation, avoiding additional branch-related computational overhead.

**Figure 3:**
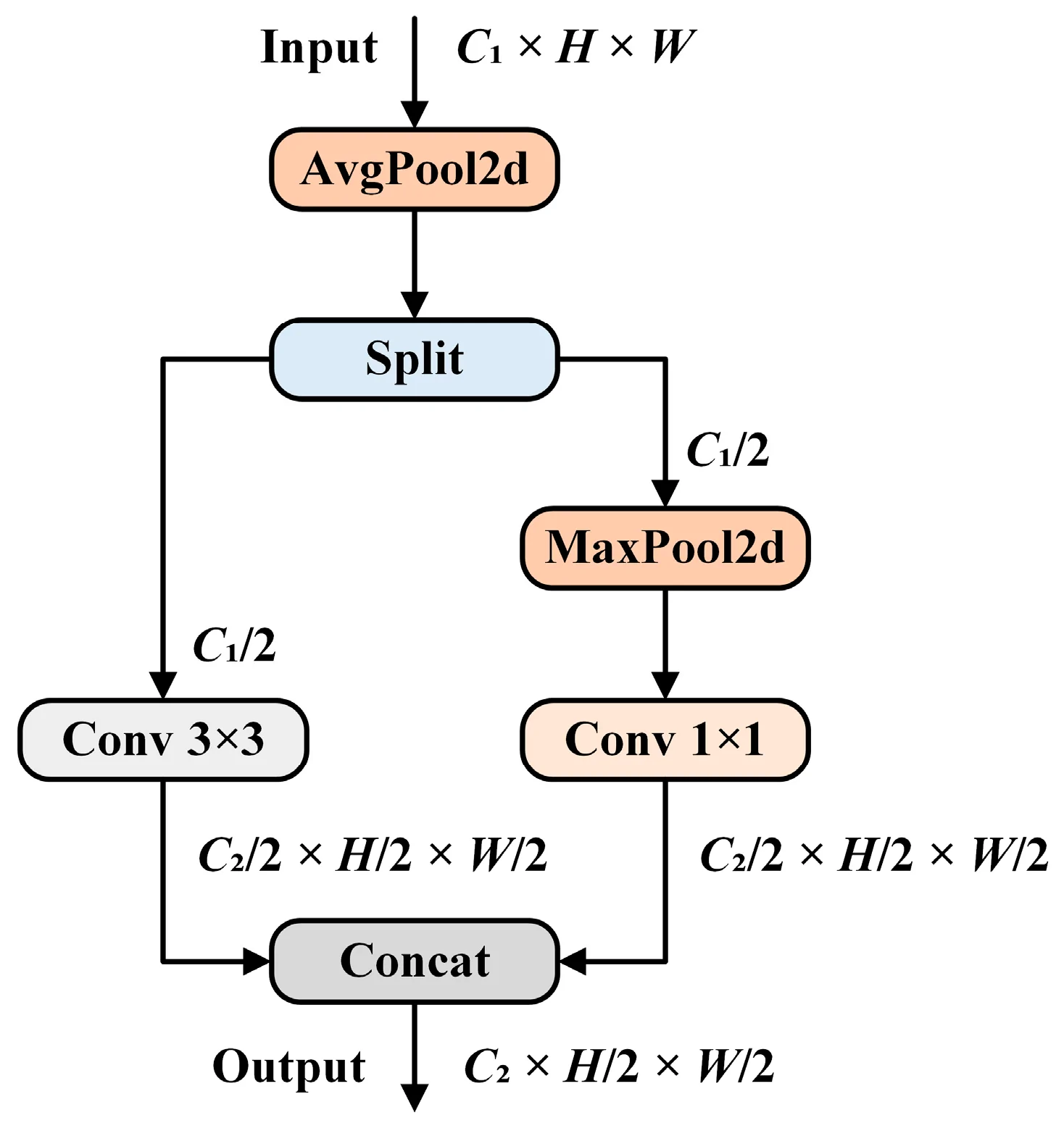
Architecture of the ADown module.

ADown modules are introduced between adjacent backbone stages for spatial downsampling, as illustrated in Figure 3. The input feature is first processed by average pooling and then divided into two channel-wise branches. The first branch applies a 3 × 3 convolution for spatial transformation, whereas the second branch uses max pooling followed by a 1 × 1 convolution for channel adjustment. The outputs of the two branches are concatenated to generate the downsampled feature representation. This design combines convolution-based transformation with pooling-based information preservation.

Together with RepNCSPELAN4, the ADown modules progressively generate the hierarchical P3, P4, and P5 backbone feature maps.

Beyond the backbone, RepNCSPELAN4 blocks are further introduced into the hybrid encoder. The original top-down and bottom-up cross-scale interaction pathway of RT-DETR is retained, while the original RepC3 fusion blocks are replaced with RepNCSPELAN4 modules. In the top-down pathway, RepNCSPELAN4 refines concatenated high-level and lower-level features after upsampling. In the bottom-up pathway, it further processes downsampled features together with the corresponding lateral features. The resulting multi-scale representations are subsequently delivered to the query selection module and RT-DETR decoder for classification and bounding box prediction.

### 3.3. Efficient Channel Attention

Bridge surface defect images often contain complex background patterns caused by concrete textures, illumination variations, shadows, water stains, and other environmental factors. Moreover, different defect categories usually exhibit variations in texture patterns, spatial structures, and appearance characteristics. Although convolutional layers can effectively extract local spatial features, they have limited capability in explicitly modeling the importance of different feature channels. Therefore, this study introduces the Efficient Channel Attention (ECA) module [28] after the P3, P4, and P5 feature outputs to construct a multi-level channel enhancement mechanism.

**Figure 4:**
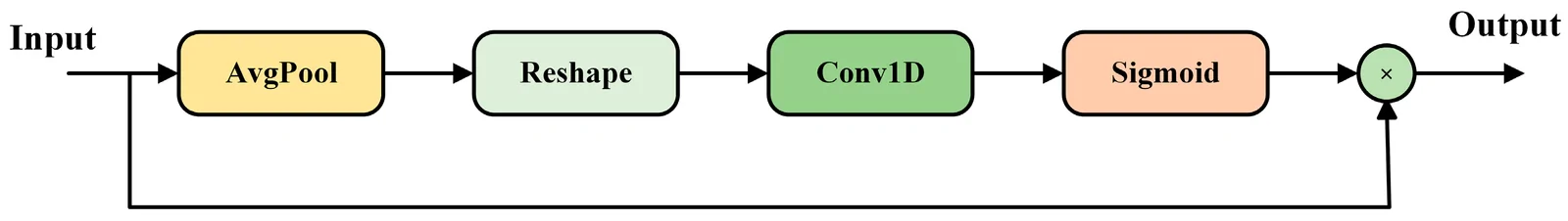
Structure of the ECA module.

The ECA module aims to adaptively recalibrate channel responses through lightweight cross-channel interaction. The structure of ECA is illustrated in Figure 4.

Given an input feature map *X* ∈ ℝ*^C^*^×*H*×*W*^, where *C*, *H*, and *W* represent the channel number, height, and width of the feature map, respectively, ECA first aggregates spatial information through global average pooling. The channel descriptor is calculated as:

(1) $$z_{c}=\frac{1}{H\times W}\sum_{i=1}^{H}\sum_{j=1}^{W}X_{c}\left(i,j\right)$$

where *z_c_* represents the global response of the *c*-th channel. Unlike traditional channel attention methods that introduce fully connected layers for channel compression and expansion, ECA directly performs local cross-channel interaction through one-dimensional convolution. The channel attention weights are obtained as:

*ω* = σ(Conv1D*_k_*(*z*)) (2)

where Conv1D*_k_*(⋅) denotes one-dimensional convolution with kernel size *k*, *σ*(⋅) represents the Sigmoid activation function, and *ω* denotes the generated channel importance weights.

The final output feature is obtained by channel-wise multiplication between the input feature map and the attention weights:

*Y* = *X* ⊗ *ω* (3)

where ⊗ denotes channel-wise feature recalibration. Through this operation, ECA enhances informative feature channels while maintaining the original spatial feature structure.

Compared with fully connected channel-attention mechanisms, ECA avoids channel dimensionality reduction and introduces only limited parameter and computational overhead. This property allows it to be embedded into the P3, P4, and P5 feature outputs for lightweight multi-level channel enhancement. In the adopted implementation, the kernel size of the one-dimensional convolution is adaptively determined according to the number of input channels as follows:

(4) $$k^{\prime }=\left|\frac{\log_{2}\left(C\right)}{\gamma }+b\right|$$

where *C* represents the number of input channels. In this study, *b* = 1 and *γ* = 2 are adopted. The obtained value is further adjusted to an odd integer to ensure symmetric local channel interaction. This adaptive strategy enables ECA to automatically select an appropriate interaction range for different feature levels, allowing the P3, P4, and P5 feature representations to obtain channel-dimension-adaptive enhancement.

### 3.4. Wise-IoU v3 for Dynamic-Focusing Bounding Box Regression

Bridge surface defects frequently exhibit irregular spatial extents, low texture contrast, and partial occlusion, leading to considerable variations in regression quality among matched predictions. RT-DETR-R50 optimizes bounding boxes using a combination of L1 and GIoU losses; however, GIoU does not explicitly differentiate regression contributions according to localization quality. To address this issue, Wise-IoU v3 [29] is introduced to replace the original GIoU term in the localization objective. Its non-monotonic dynamic focusing mechanism suppresses excessive contributions from extremely low-quality predictions, reduces the dominance of predictions that are already well localized, and places greater emphasis on intermediate-quality predictions with meaningful optimization potential. Because this modification affects only the training objective, it introduces no additional parameters, FLOPs, or inference latency.

The IoU loss between a predicted box and its matched ground-truth box is first defined as

*L*_IoU_ = 1 − IoU (5)

To account for the distance between their center points, Wise-IoU introduces the following distance attention term:

(6) $$R_{\mathrm{WIoU}}=\exp\left(\frac{{\left(x-x_{\mathrm{gt}}\right)}^{2}+{\left(y-y_{\mathrm{gt}}\right)}^{2}}{{\left(W_{g}^{2}+H_{g}^{2}\right)}^{*}}\right),$$

where *x* and *y* denote the center coordinates of the predicted box, whereas *x*_gt_ and *y*_gt_ denote those of the ground-truth box. *W_g_* and *H_g_* are the width and height of the minimum enclosing box. The superscript (⋅)* indicates that the corresponding term is detached from the computational graph. The basic Wise-IoU loss is then expressed as

(7) $$L_{\mathrm{WIoU}}^{v1}=R_{\mathrm{WIoU}}L_{\mathrm{IoU}}$$

Wise-IoU v3 further introduces an outlier degree to characterize the relative quality of each matched prediction:

(8) $$\beta =\frac{L_{\mathrm{IoU}}^{*}}{{\hat{L}}_{\mathrm{IoU}}},$$

where $L_{\mathrm{IoU}}^{*}$ is detached from gradient propagation and ${\hat{L}}_{\mathrm{IoU}}$ is the running mean of the IoU loss. In the implementation, ${\hat{L}}_{\mathrm{IoU}}$ is initialized to 1.0 and updated at each loss evaluation as

(9) $${\hat{L}}_{\mathrm{IoU}}^{\left(t\right)}=\left(1-m\right){\hat{L}}_{\mathrm{IoU}}^{\left(t-1\right)}+m\;\mathrm{mean}\left(L_{\mathrm{IoU}}^{\left(t\right)}\right),\;\;m=0.01.$$

Based on the outlier degree, the non-monotonic focusing coefficient is defined as

(10) $$r=\frac{\beta }{\delta \alpha^{\beta -\delta }},$$

where *α* = 1.7 and *δ* = 2.7 control the shape of the focusing curve. The resulting Wise-IoU v3 is therefore defined as

(11) $$L_{\mathrm{WIoU}}^{v3}=rL_{\mathrm{WIoU}}^{v1}$$

In the implementation, the non-monotonic mode is enabled by setting monotonous = False, while the Inner-IoU and Focaler-IoU extensions are disabled. The L1 and Wise-IoU v3 terms are assigned weights of 5 and 2, respectively. The resulting bounding box regression loss is

(12) $$L_{\mathrm{box}}=5L_{1}+2L_{\mathrm{WIoU}}^{v3}$$

Wise-IoU v3 replaces the GIoU term in the main decoder, auxiliary decoder, and denoising localization losses. The classification loss, Hungarian matching mechanism, and remaining auxiliary and denoising loss structures are retained. The resulting objective redistributes regression gradients to improve bounding box localization for defects with irregular spatial extents or ambiguous box annotations.

## 4. Experimental Results and Analysis

### 4.1. Experimental Setup

All experiments were conducted on a Linux server equipped with an Intel Xeon Silver 4110 CPU, 251 GB RAM, and four NVIDIA GeForce RTX 2080 Ti GPUs. The operating system was Ubuntu 16.04.7 LTS with CUDA 11.3. Model training was performed using all four GPUs, whereas the end-to-end inference speed tests described in Section 4.3 were conducted on a single GPU. The software environment consisted of PyTorch 1.13.0 and CUDA 11.3. The RT-DETR and YOLO series models were implemented using an Ultralytics-based codebase under Python 3.7.4, while Faster R-CNN and RetinaNet were implemented using MMDetection under Python 3.9. All models were trained from scratch without pretrained weights.

Within each dataset, all compared models used identical training, validation, and test splits with an input resolution of 640 × 640 pixels. Training was conducted for a maximum of 600 epochs with four data-loading workers and an early-stopping patience of 50 epochs. The global batch size was set to 16 across four GPUs, corresponding to four images per GPU for MMDetection-based models. RT-DETR and REW-DETR were optimized using AdamW with an initial learning rate of 1 × 10^−4^, whereas Faster R-CNN and RetinaNet employed SGD with initial learning rates of 0.02 and 0.005, respectively. For YOLO-based models, the optimizer and learning rate were determined using the Ultralytics Auto strategy, and other training parameters followed the default configuration. The weight decay was set to 1 × 10^−4^ for manually configured models.

Due to computational resource limitations, each experiment was conducted with a fixed random seed, and repeated multi-seed evaluation was not performed.

### 4.2. Dataset Construction and Splitting Strategy

This study used the publicly available bridge surface defect dataset introduced in [30] as the primary benchmark. The released dataset contains 6308 images, including samples generated through spatial transformation and blur operations.

**Figure 5:**
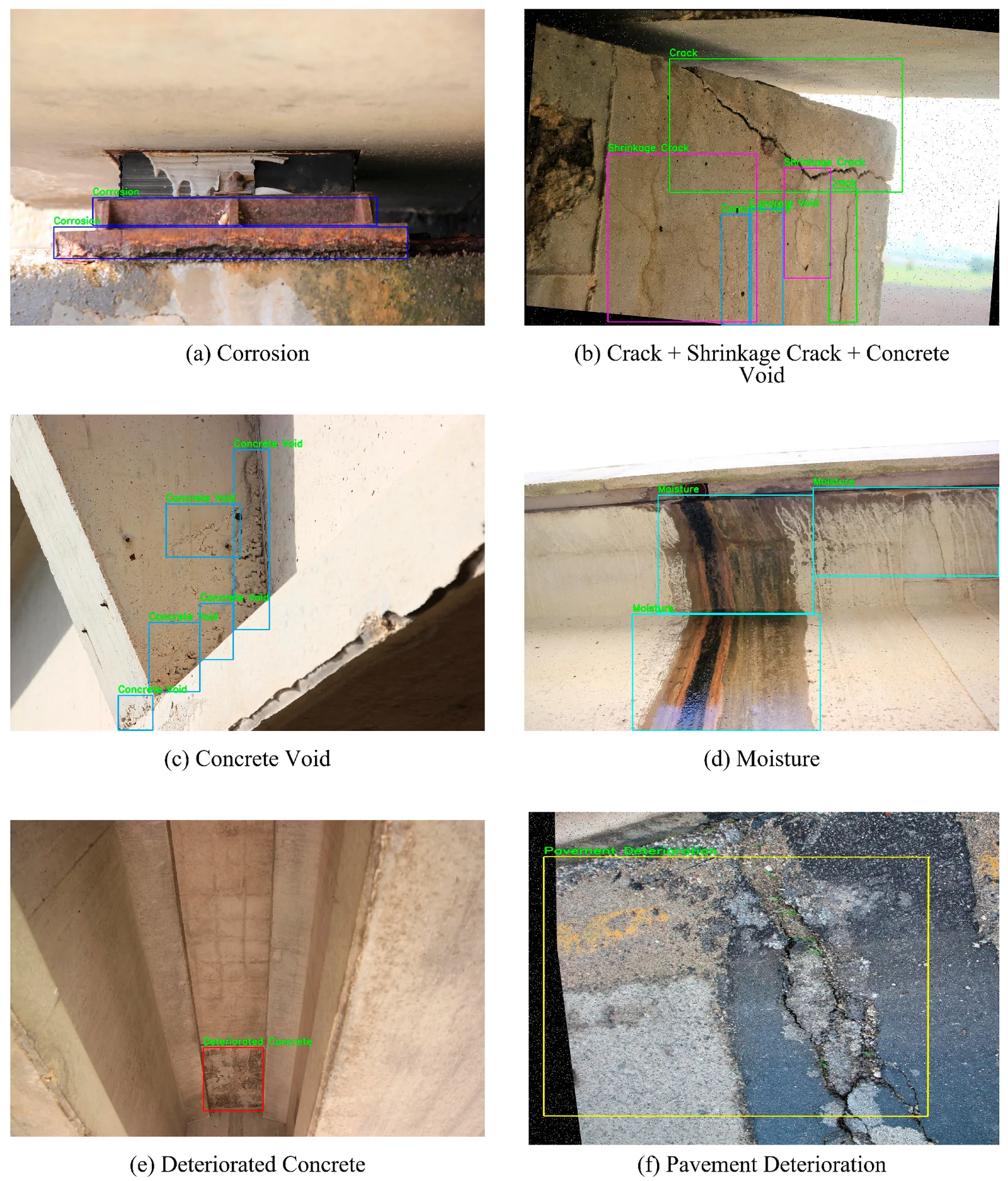
Representative annotated images from the primary bridge surface defect dataset.

The original annotations consist of eight categories: Corrosion, Crack, Deteriorated Concrete, Concrete Void, Moisture, Pavement Deterioration, Shrinkage Crack, and Bottom Shrinkage Crack. Since Bottom Shrinkage Crack contains limited samples and exhibits strong visual similarity to Shrinkage Crack, these two categories were merged. The final dataset contains seven defect categories, and representative examples are presented in Figure 5.

After category merging and annotation organization, the dataset contains 55,498 defect bounding boxes. The category distribution is summarized in Table 1. Corrosion and Moisture contain 22,518 and 15,058 annotations, respectively, whereas Pavement Deterioration and Shrinkage Crack contain only 77 and 171 annotations. This distribution indicates a significant class imbalance and provides a challenging scenario for evaluating rare defect detection. No dedicated class-balanced sampling, class-specific reweighting, or additional imbalance-oriented classification loss was introduced in the REW-DETR training pipeline; the original category distribution of the fixed training split was retained during training.

Because transformed images and continuously captured images may remain visually similar, directly performing random image-level splitting may introduce data leakage by distributing near-duplicate samples into different subsets. To reduce this risk, perceptual hashing (pHash) was adopted for image grouping. Images were grouped using the original greedy pHash procedure with a Hamming-distance threshold of 4. To evaluate the sensitivity of this threshold, the same grouping procedure was reproduced using thresholds of 2, 4, and 6. The three thresholds produced 6293, 6250, and 6131 groups, respectively, including 14, 51, and 163 multi-image groups, with corresponding maximum group sizes of 3, 7, and 9 images. When the reconstructed groups were checked against the fixed training, validation, and test split, the numbers of cross-split grouping conflicts were 0, 0, and 74 for thresholds of 2, 4, and 6, respectively. The threshold of 2 was therefore more restrictive and identified relatively few multi-image groups, whereas increasing the threshold to 6 substantially increased both the number of multi-image groups and cross-split grouping conflicts. A threshold of 4 was retained as an intermediate criterion that increased the identification of candidate near-duplicate groups without producing cross-split grouping conflicts under the original greedy grouping procedure. In addition, no visually confirmed near-duplicate image pair was found across the final training, validation, and test subsets. The resulting groups were divided into training, validation, and test sets with a target ratio of approximately 7:2:1.

Due to differences in group size, the final training, validation, and test sets contained 4413, 1258, and 637 images, corresponding to approximately 70%, 20%, and 10% of the dataset, respectively. The training set was used for model optimization, the validation set for model selection and training monitoring, and the test set was reserved for final performance evaluation. All subsequent attention module selection, ablation studies, comparative experiments, class-wise analyses, and visualization experiments were conducted using this fixed split.

### 4.3. Performance Metrics

To evaluate REW-DETR from different perspectives, this study considers detection accuracy, model complexity, and inference efficiency. Detection performance is evaluated using Precision, Recall, mAP_50_, and mAP_50:95_ [31]. Model complexity is measured by the number of parameters (Params) and floating-point operations (FLOPs), while inference efficiency is evaluated using end-to-end frames per second (FPS).

Precision and Recall are defined as follows:

(13) $$\mathrm{Precision}=\frac{\mathrm{TP}}{\mathrm{TP}+\mathrm{FP}}$$

(14) $$\mathrm{Recall}=\frac{\mathrm{TP}}{\mathrm{TP}+\mathrm{FN}}$$

where TP, FP, and FN denote true positives, false positives, and false negatives, respectively. Since different detection frameworks may adopt different confidence thresholds and post-processing strategies, mAP is mainly used for cross-model comparison, while Precision and Recall are reported as complementary metrics.

Average Precision (AP) is calculated as the area under the Precision-Recall curve:

(15) $$\mathrm{AP}=\int_{0}^{1}P(R)\mathrm{dR}$$

where P(R) represents the Precision value at different Recall levels. For multi-class defect detection, mean Average Precision (mAP) is calculated as:

(16) $$\mathrm{mAP}=\frac{1}{N}\sum_{i=1}^{N}AP_{i}$$

where N denotes the number of defect categories and AP_i_ represents the AP of the i-th category under a specified IoU threshold. This study reports mAP_50_ and mAP_50:95_. The mAP_50_ metric is calculated at an IoU threshold of 0.5, whereas mAP_50:95_ averages AP values over IoU thresholds from 0.5 to 0.95 with an interval of 0.05, providing a stricter evaluation of localization quality.

Model complexity is evaluated using Params and FLOPs. Params represents the number of learnable parameters, while FLOPs measures the computational cost of a single forward pass. To ensure consistent comparison among different frameworks, FLOPs are calculated using a batch size of 1, FP32 precision, and an input tensor of 1 × 3 × 640 × 640. A deterministic input tensor is used instead of real images to avoid variations caused by image content, aspect ratio, and framework-specific preprocessing procedures.

Inference efficiency is measured using end-to-end FPS on a single NVIDIA GeForce RTX 2080 Ti GPU with an input resolution of 640 × 640. The batch size was fixed to 4 to ensure stable GPU utilization during throughput measurement. FPS includes image preprocessing, model inference, and detection post-processing, but excludes disk reading and data-loading time. Although the same GPU, input resolution, batch size, and end-to-end timing scope were used for all models, the different codebases retain framework-specific preprocessing, post-processing, and software execution paths. Therefore, the reported FPS should be interpreted as implementation-dependent throughput under the adopted experimental environment rather than as a strictly framework-neutral measure of network latency. Small FPS differences between models implemented in different frameworks should consequently be interpreted with caution.

### 4.4. Experimental Results

#### 4.4.1. Attention Mechanism and Placement Comparison

To identify an attention mechanism suitable for bridge surface defect detection, CoordAtt [32], SimAM [33], EMA [34], TripletAtt [35], and ECA [28] were evaluated on the RT-DETR-R50 validation set. For a controlled comparison, all candidate modules were first inserted after P5, while ECA was additionally evaluated at P3, P4, and P5. The results are reported in Table 2.

CoordAtt, SimAM, and EMA produced only limited improvements over the baseline, whereas TripletAtt reduced both mAP_50_ and mAP_50:95_. In contrast, single-level ECA increased mAP_50_ from 65.6% to 69.8% and mAP_50:95_ from 36.1% to 38.9% without materially changing Params or FLOPs. Extending ECA to P3, P4, and P5 further increased mAP_50_ and mAP_50:95_ to 70.1% and 40.3%, respectively. Although FPS decreased from 52.2 to 50.1, the computational overhead remained limited. These results indicate that multi-level ECA provides the most favorable accuracy–efficiency trade-off among the evaluated attention configurations and was therefore adopted in the subsequent experiments.

#### 4.4.2. Ablation Study

**Figure 6:**
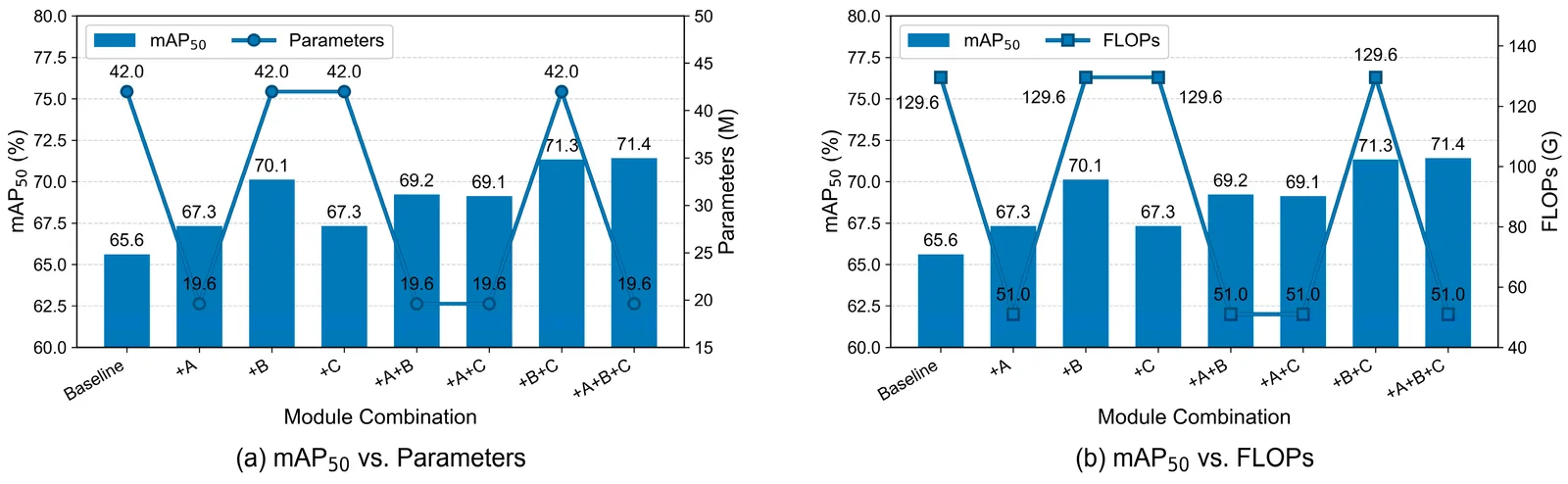
Accuracy–complexity comparison of different ablation configurations on the validation set. (**a**) mAP_50_ and parameter count; (**b**) mAP_50_ and FLOPs. A denotes the RepNCSPELAN4–ADown backbone and RepNCSPELAN4-based multi-scale fusion architecture, B denotes the multi-level ECA scheme, and C denotes Wise-IoU v3.

To evaluate the individual and combined contributions of the proposed components, controlled ablation experiments were conducted on the validation set. Module A denotes the RepNCSPELAN4–ADown backbone and RepNCSPELAN4-based fusion architecture, module B denotes multi-level ECA at P3, P4, and P5, and module C denotes Wise-IoU v3. The results are presented in Table 3 and Figure 6.

Module A produced the main reduction in model complexity. It decreased Params from 42.0 M to 19.6 M and FLOPs from 129.6 G to 51.0 G, while increasing FPS from 52.2 to 73.1 and slightly improving both mAP metrics. Module B provided the largest individual accuracy gain, increasing mAP_50_ and mAP_50:95_ by 4.5 and 4.2 percentage points, respectively, with negligible changes in Params and FLOPs. Module C also improved localization performance without modifying the inference structure.

The combined results indicate that the interactions among the three modules are metric-dependent rather than strictly additive. Adding module C increased mAP_50_ in every evaluated context: from 65.6% to 67.3% on the baseline, from 67.3% to 69.1% when combined with A, from 70.1% to 71.3% when combined with B, and from 69.2% to 71.4% when added to A+B. In contrast, its effect on mAP_50:95_ varied across configurations. Module C increased mAP_50:95_ from 36.1% to 38.2% on the baseline and from 39.4% to 39.8% on A+B, but reduced it from 37.8% to 36.1% on A and from 40.3% to 39.9% on B. This behavior indicates that the regression optimization introduced by Wise-IoU v3 does not provide an independent additive gain under all feature configurations, particularly under the stricter IoU thresholds represented by mAP_50:95_. Module B remained the strongest individual contributor to mAP_50:95_, whereas module A provided the dominant reduction in model complexity. The complete A+B+C configuration achieved the highest mAP_50_ of 71.4% with 19.6 M parameters, 51.0 G FLOPs, and 73.0 FPS, while its mAP_50:95_ of 39.8% was 0.5 percentage points lower than B alone and 0.1 percentage points lower than B+C. Therefore, the final architecture was selected based on the overall accuracy–complexity trade-off rather than the maximum mAP_50:95_ alone.

#### 4.4.3. Comparison with Representative Detectors

The final REW-DETR model was compared with representative two-stage, YOLO-based, and RT-DETR-based detectors on the fixed test split. All models were evaluated at a nominal input resolution of 640 × 640 under the hardware and framework-specific evaluation settings described in Section 4.1 and Section 4.3. The results are reported in Table 4.

REW-DETR achieved a Precision of 87.0%, a Recall of 70.4%, an mAP_50_ of 73.7%, and an mAP_50:95_ of 42.4%, representing the highest values among the compared models. Relative to RT-DETR-R50, these metrics increased by 3.8, 5.5, 4.0, and 4.5 percentage points, respectively. At the same time, Params decreased from 42.0 M to 19.6 M and FLOPs from 129.6 G to 51.0 G, corresponding to reductions of approximately 53.3% and 60.6%. End-to-end FPS increased from 51.2 to 70.8.

The YOLO models generally provided higher throughput but lower mAP values, whereas the evaluated RT-DETR variants generally achieved higher mAP values than the YOLO models, although the larger RT-DETR variants incurred greater computational cost. REW-DETR did not obtain the highest FPS among all models, but it combined the strongest detection results with substantially lower complexity than RT-DETR-R50. Its principal advantage therefore lies in the overall accuracy–efficiency trade-off rather than throughput alone. Because the compared detector families use different framework-specific optimization and training strategies, the absolute accuracy differences in Table 4 may reflect both architectural characteristics and implementation-specific training recipes. Similarly, although inference speed was evaluated using the same GPU, input resolution, batch size, and end-to-end timing scope, different codebases retain framework-specific preprocessing, post-processing, and software execution paths. Therefore, Table 4 should be interpreted as a practical comparison of representative implementations under matched dataset, input-resolution, and hardware conditions rather than as a strictly optimizer- and framework-controlled architecture-only benchmark. Small cross-framework differences in accuracy or FPS should consequently be interpreted with caution. In contrast, RT-DETR-R50 and REW-DETR share the same implementation framework and training protocol, providing a more directly controlled comparison for evaluating the effects of the proposed modifications.

#### 4.4.4. Category-Wise Performance Analysis

Table 5 compares the category-level performance of RT-DETR-R50 and REW-DETR on the test set. REW-DETR improved the overall Precision, mAP_50_, and mAP_50:95_ by 3.8, 4.0, and 4.5 percentage points, respectively.

The largest mAP_50_ gains were obtained for Crack, Concrete Void, and Moisture, with improvements of 8.6, 6.4, and 6.3 percentage points. Their mAP_50:95_ values also increased by 5.4, 5.5, and 5.2 percentage points, respectively. These categories contain slender, irregular, low-contrast, or background-sensitive defects, suggesting that the proposed feature enhancement and regression optimization are particularly beneficial under visually ambiguous conditions. Smaller gains were observed for Corrosion and Deteriorated Concrete.

Despite the improvements over RT-DETR-R50, Crack and Corrosion still achieved relatively low mAP_50:95_ values of 32.3% and 29.4%, respectively, compared with their mAP_50_ values of 63.7% and 61.9%. This gap indicates that these categories remain more sensitive to stricter localization criteria. For Crack, the slender geometry, small spatial extent, weak contrast, and indistinct boundaries make precise bounding-box localization difficult, because small deviations in box position or size can substantially reduce IoU for narrow targets. Corrosion, in contrast, often exhibits irregular and spatially diffuse regions with heterogeneous surface appearance, making the exact defect extent less visually distinct. Consequently, predicted boxes may cover the correct general region while differing from the annotated extent under higher IoU thresholds. These results suggest that precise localization of slender, weak-boundary, and spatially diffuse defects remains a limitation of the current model.

**Figure 7:**
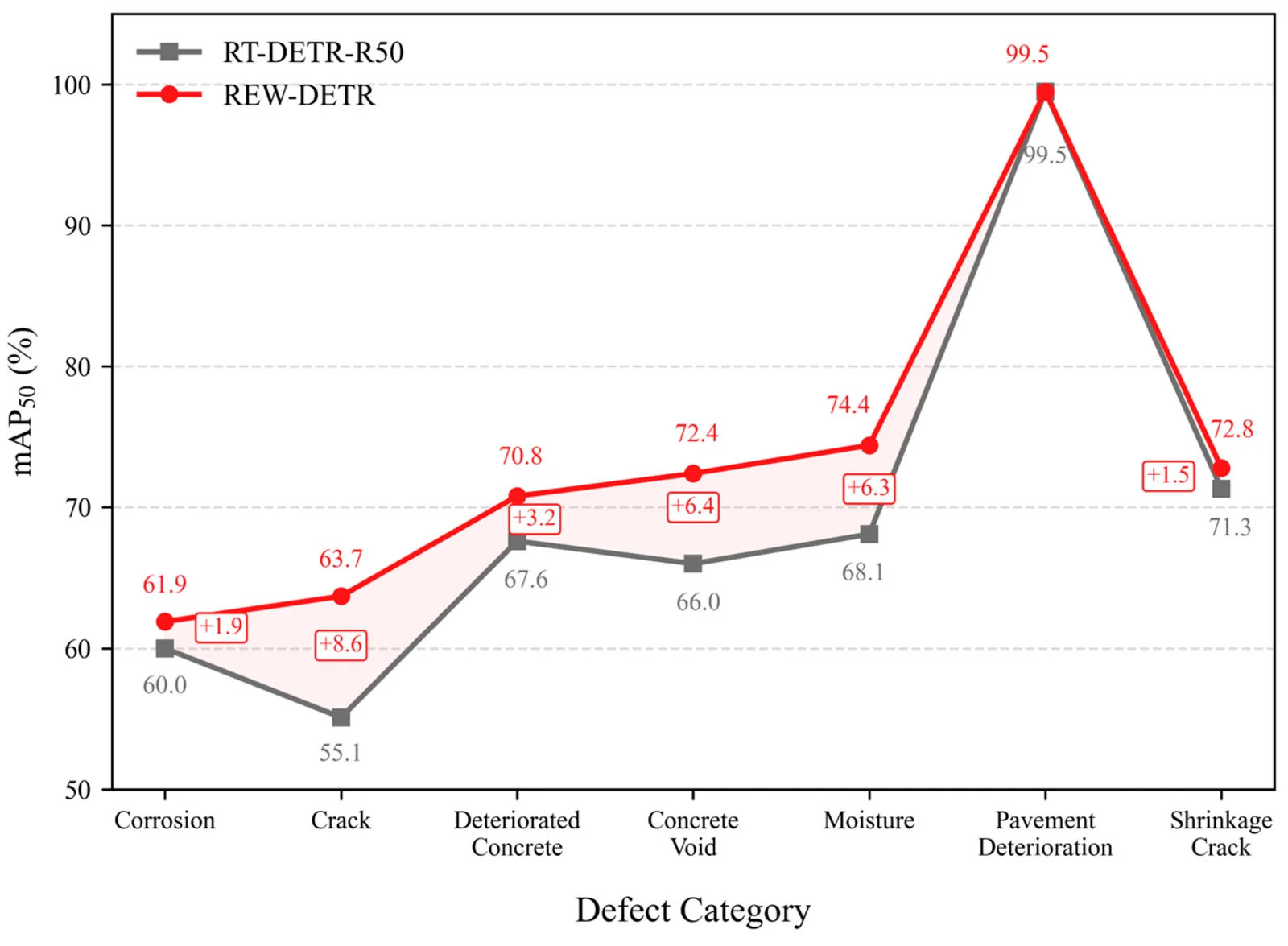
Class-wise comparison of mAP_50_ between RT-DETR-R50 and REW-DETR on the test set.

For Shrinkage Crack, Precision decreased from 85.4% to 83.5%, while mAP_50_ and mAP_50:95_ increased from 71.3% and 43.6% to 72.8% and 50.2%, respectively. However, Shrinkage Crack and Pavement Deterioration contain only 171 and 77 annotations in the complete dataset, accounting for approximately 0.31% and 0.14% of the 55,498 annotations, respectively. In comparison, Corrosion contains 22,518 annotations, approximately 132 and 292 times the numbers available for Shrinkage Crack and Pavement Deterioration, respectively. Consequently, the class-wise AP values of these rare categories should be interpreted cautiously because the limited number of annotated instances may make their performance estimates more sensitive to a small number of correct or missed detections. In particular, the 99.5% mAP_50_ obtained for Pavement Deterioration should not be interpreted as having the same stability as the AP estimates of well-represented categories. Future work could investigate class-aware sampling, loss reweighting, and targeted augmentation for rare categories to further reduce the influence of the observed class imbalance. Overall, REW-DETR improved mAP_50_ in six of the seven categories and improved mAP_50:95_ across all seven categories, while rare classes and categories with substantial appearance variation remain challenging. The category-wise mAP_50_ trends of the two models are further visualized in Figure 7.

#### 4.4.5. Qualitative Detection Results

**Figure 8:**
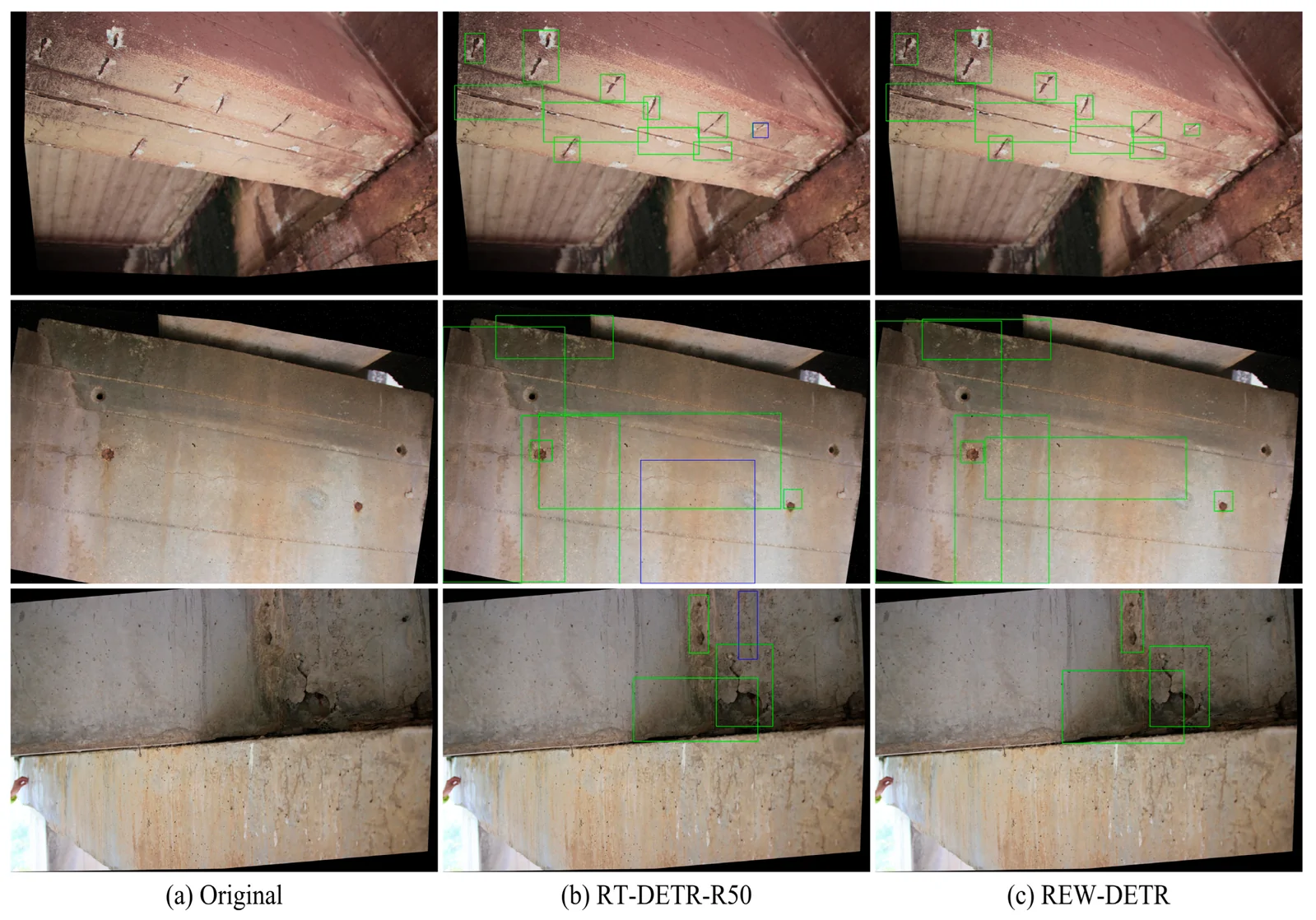
Qualitative comparison of detection results produced by RT-DETR-R50 and REW-DETR on selected test images. (**a**) Original images; (**b**) RT-DETR-R50 predictions; (**c**) REW-DETR predictions. Green boxes denote detections matched to the ground-truth annotations, whereas blue boxes indicate false-positive predictions.

To examine qualitative differences between the two models, several images from the bridge surface defect test set were selected to illustrate typical detection outcomes, as shown in Figure 8. The original images are presented in Figure 8a, while Figure 8b and Figure 8c show the predictions produced by RT-DETR-R50 and REW-DETR, respectively. Green boxes denote detections matched to the ground-truth annotations, whereas blue boxes indicate false-positive predictions.

In the selected examples, RT-DETR-R50 correctly identified most prominent defects but produced additional predictions around concrete joints, structural edges, and regions with strong local texture variation. These errors were more apparent in scenes containing small targets, weak contrast, or visually complex backgrounds. REW-DETR retained the principal correct detections while reducing several background-related false positives, resulting in cleaner prediction outputs in these cases.

Because qualitative results depend on the selected images, Figure 8 is intended only to illustrate differences in false-positive behavior and background sensitivity between the two models. Conclusions regarding overall detection performance should primarily be based on the quantitative results reported in Table 4 and Table 5.

#### 4.4.6. Grad-CAM++ Analysis

**Figure 9:**
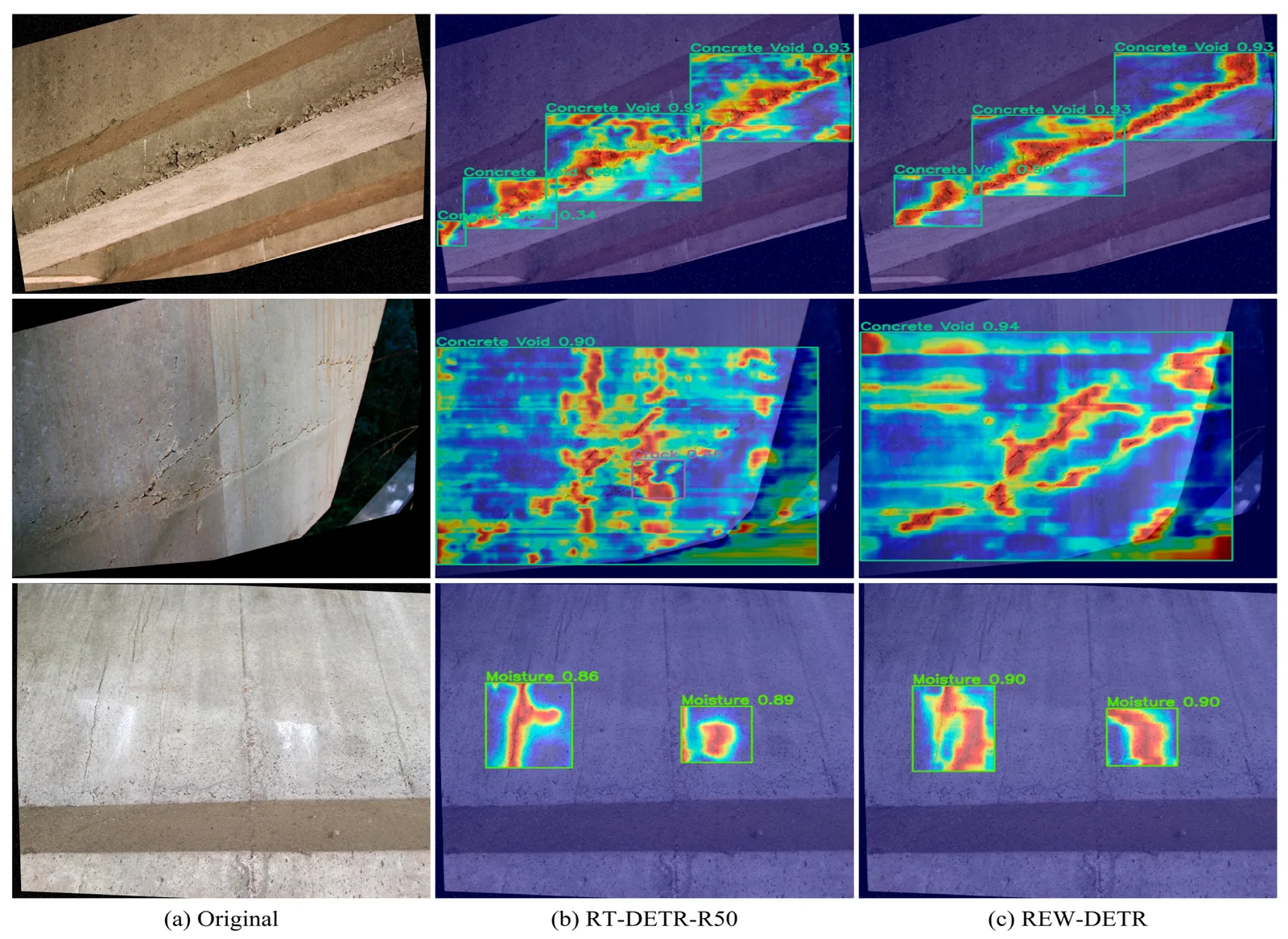
Grad-CAM++ comparison of RT-DETR-R50 and REW-DETR on selected test images. (**a**) Original images; (**b**) RT-DETR-R50 response heatmaps; (**c**) REW-DETR response heatmaps.

To examine the spatial regions emphasized by the two detectors, Grad-CAM++ was applied to generate response heatmaps for bridge surface defect predictions. For visualization, the response values within each predicted bounding box were normalized. Warmer colors indicate stronger model responses, whereas cooler colors represent weaker responses. Concrete Void and Moisture examples were selected from the test set for qualitative comparison. Figure 9a presents the original images, while Figure 9b and Figure 9c show the Grad-CAM++ results of RT-DETR-R50 and REW-DETR, respectively.

In the selected examples, the high-response regions produced by REW-DETR were more closely concentrated around the visible defect areas and showed less activation over adjacent concrete textures and non-defect backgrounds. By comparison, RT-DETR-R50 generated broader or more fragmented response regions in several cases, and its corresponding detection outputs included redundant predictions or incorrect category assignments. REW-DETR placed a larger proportion of its response around the defect body and nearby boundary regions, indicating more localized defect-related activation in these examples.

These visualizations provide qualitative support for interpreting the different response patterns of the two models. However, Grad-CAM++ is an auxiliary interpretability tool rather than a quantitative performance measure. The observed heatmap patterns depend on the selected samples and should therefore be considered together with the complete test-set results reported in Table 4 and Table 5.

### 4.5. Evaluation on an Additional Bridge Defect Dataset

Supplementary experiments were conducted on the bridge-defects-detection dataset from Roboflow Universe [43] to evaluate REW-DETR after retraining under a different data distribution. The adopted version contains 4459 images divided into 3121 training, 892 validation, and 446 test images. The original five-class setting was retained, including the source-provided for-review label, although this label was not interpreted as a specific physical defect category. All models were retrained using the same split, input resolution, and hardware environment.

As shown in Table 6, REW-DETR achieved the highest Recall and mAP_50_, reaching 58.0% and 55.4%, respectively. Compared with RT-DETR-R50, Recall and mAP_50_ increased by 1.7 and 0.8 percentage points, while Params and FLOPs decreased by approximately 53.3% and 60.6%. FPS increased from 43.5 to 55.8.

The mAP_50_ difference between REW-DETR and YOLOv8m was only 0.1 percentage points, indicating comparable rather than decisive accuracy superiority. The main advantage of REW-DETR on this dataset is therefore its combination of competitive detection performance, the highest Recall, and low computational complexity. Because all models were retrained on the additional dataset, these results demonstrate adaptability after dataset-specific training rather than cross-dataset generalization without adaptation.

## 5. Conclusions

This study proposed REW-DETR, a lightweight end-to-end detector for bridge surface defects, to address large target-scale variation, complex background interference, and the high computational cost of RT-DETR-R50. The proposed framework jointly redesigns lightweight feature extraction and multi-scale fusion using RepNCSPELAN4–ADown, introduces multi-level ECA for channel enhancement, and adopts Wise-IoU v3 for bounding box regression. The ablation results confirmed that these components make distinct contributions to detection accuracy and computational efficiency.

On the primary bridge surface defect test set, REW-DETR achieved a Precision of 87.0%, a Recall of 70.4%, an mAP_50_ of 73.7%, and an mAP_50:95_ of 42.4%. Compared with RT-DETR-R50, these metrics increased by 3.8, 5.5, 4.0, and 4.5 percentage points, respectively. Meanwhile, the parameter count and FLOPs were reduced from 42.0 M and 129.6 G to 19.6 M and 51.0 G, corresponding to reductions of approximately 53.3% and 60.6%, while end-to-end FPS increased from 51.2 to 70.8. Category-wise evaluation showed particularly clear improvements for Crack, Concrete Void, and Moisture.

After dataset-specific retraining on the additional bridge-defects-detection dataset, REW-DETR achieved a Recall of 58.0% and an mAP_50_ of 55.4% while retaining its lightweight configuration. These results provide supplementary evidence that the model remains competitive under a different image source, category definition, and sample distribution. However, because the model was retrained on the additional dataset, this experiment demonstrates adaptability rather than cross-dataset generalization without adaptation.

Overall, REW-DETR provides a favorable balance among detection accuracy, model complexity, and inference efficiency, providing a basis for further evaluation of the model in UAV-assisted bridge inspection and other resource-constrained scenarios. Nevertheless, onboard inference and field deployment on UAVs or edge devices have not yet been evaluated, and performance for rare categories and challenging localization conditions requires further improvement. In addition, the present experiments do not separately quantify robustness to illumination variation, motion blur, viewing distance, camera angle, occlusion, or image-resolution changes. Future work will therefore include controlled condition-specific robustness evaluation under these UAV-relevant imaging factors and broader bridge environments, while investigating model pruning, quantization, hardware-aware optimization, and embedded-device acceleration.

## Figures and Tables

**Table 1:** Category-wise distribution of defect bounding boxes in the dataset.

Defect Category	Number of Bounding Boxes
Corrosion	22,518
Crack	3085
Deteriorated Concrete	6862
Concrete Void	7727
Moisture	15,058
Pavement Deterioration	77
Shrinkage Crack	171
Total	55,498

**Table 2:** Validation-set performance comparison of different attention mechanisms and ECA placement strategies on the RT-DETR-R50 baseline, where "√" indicates the use of the corresponding mechanism at the specific feature level (P3, P4, or P5).

Model	P3	P4	P5	Precision (%)	mAP_50_ (%)	mAP_50:95_ (%)	Parameters (M)	FLOPs (G)	FPS
Baseline				80.4	65.6	36.1	42.0	129.6	52.2
+CoordAtt			**√**	82.3	66.3	37.0	42.0	129.6	52.1
+SimAM			**√**	82.3	66.3	36.7	42.0	129.6	52.1
+EMA			**√**	82.7	66.3	36.9	42.0	129.6	51.3
+TripletAtt			**√**	71.2	57.5	30.4	42.0	129.6	50.6
+ECA			**√**	84.8	69.8	38.9	42.0	129.6	52.3
+ECA	**√**	**√**	**√**	87.4	70.1	40.3	42.0	129.6	50.1

**Table 3:** Results of the ablation experiment, where "√" indicates the use of the corresponding improvement module (A, B, or C).

Baseline	A	B	C	mAP_50_ (%)	mAP_50:95_ (%)	Parameters (M)	FLOPs (G)	FPS
RT-DETR-R50				65.6	36.1	42.0	129.6	52.2
	**√**			67.3	37.8	19.6	51.0	73.1
		**√**		70.1	40.3	42.0	129.6	50.1
			**√**	67.3	38.2	42.0	129.6	52.5
	**√**	**√**		69.2	39.4	19.6	51.0	73.6
	**√**		**√**	69.1	36.1	19.6	51.0	73.5
		**√**	**√**	71.3	39.9	42.0	129.6	52.5
	**√**	**√**	**√**	71.4	39.8	19.6	51.0	73.0

**Table 4:** Performance comparison between REW-DETR and representative convolutional and Transformer-based object detectors on the bridge surface defect test set.

Model	Precision (%)	Recall (%)	mAP_50_ (%)	mAP_50:95_ (%)	Parameters (M)	FLOPs (G)	FPS
Convolution-based object detection models							
Faster R-CNN [36]	58.9	37.3	38.2	14.3	41.4	90.9	43.1
RetinaNet [37]	36.6	41.6	37.9	14.8	36.5	82.6	54.8
YOLOv8m [38]	80.9	57.8	65.1	33.2	25.8	78.7	110.1
YOLOv9m [27]	76.9	57.2	63.8	34.9	20.0	76.5	97.1
YOLOv10b [39]	71.5	57.3	63.0	34.1	19.0	91.6	98.1
YOLO11m [40]	85.3	54.5	65.3	35.6	20.0	67.7	105.0
YOLO12m [41]	78.0	56.3	64.4	33.0	20.1	67.1	89.5
YOLO26m [42]	82.8	56.5	64.3	34.6	20.4	67.9	114.5
Transformer-based object detection models							
RT-DETR-R18	80.5	63.9	69.2	38.3	19.9	57.0	96.3
RT-DETR-R34	81.2	63.6	69.3	39.1	31.1	88.8	73.2
RT-DETR-L	81.5	64.5	69.4	39.5	32.0	103.5	59.0
RT-DETR-R50	83.2	64.9	69.7	37.9	42.0	129.6	51.2
REW-DETR	87.0	70.4	73.7	42.4	19.6	51.0	70.8

**Table 5:** Category-wise detection performance of RT-DETR-R50 and REW-DETR on the bridge surface defect test set.

Model	Defect Category	Precision (%)	mAP_50_ (%)	mAP_50:95_ (%)
RT-DETR-R50	All	83.2	69.7	37.9
	Corrosion	78.1	60.0	28.6
	Crack	80.2	55.1	26.9
	Deteriorated Concrete	83.7	67.6	38.5
	Concrete Void	76.6	66.0	34.3
	Moisture	82.4	68.1	38.4
	Pavement Deterioration	95.9	99.5	54.8
	Shrinkage Crack	85.4	71.3	43.6
REW-DETR	All	87.0	73.7	42.4
	Corrosion	78.9	61.9	29.4
	Crack	85.4	63.7	32.3
	Deteriorated Concrete	87.3	70.8	39.9
	Concrete Void	88.5	72.4	39.8
	Moisture	88.7	74.4	43.6
	Pavement Deterioration	96.9	99.5	61.3
	Shrinkage Crack	83.5	72.8	50.2

**Table 6:** Detection performance and computational complexity of representative object detectors on the bridge-defects-detection test set.

Model	Precision (%)	Recall (%)	mAP_50_ (%)	Parameters (M)	FLOPs (G)	FPS
Convolution-based object detection models						
Faster R-CNN	52.1	48.0	44.9	41.4	90.9	50.5
RetinaNet	52.4	48.0	43.1	36.5	82.6	64.9
YOLOv8m	56.1	57.4	55.3	25.8	78.7	123.7
YOLOv9m	56.6	55.8	55.2	20.0	76.5	102.6
YOLOv10b	56.6	56.6	54.1	19.0	91.6	115.8
YOLOv12m	57.6	55.0	54.9	20.1	67.1	103.9
Transformer-based object detection models						
RT-DETR-R18	55.0	53.1	52.6	19.9	57.0	73.2
RT-DETR-R34	56.4	54.0	54.0	31.1	88.8	61.8
RT-DETR-L	57.5	54.2	53.7	32.0	103.5	52.5
RT-DETR-R50	56.6	56.3	54.6	42.0	129.6	43.5
REW-DETR	56.0	58.0	55.4	19.6	51.0	55.8

## Data Availability

The processed data and experimental configurations supporting the conclusions of this article are available from the authors upon reasonable request, subject to the terms of the original data providers.
